# TGF-β induces HLA-G expression through inhibiting miR-152 in gastric cancer cells

**DOI:** 10.1186/s12929-015-0177-4

**Published:** 2015-12-02

**Authors:** Zhongzheng Guan, Bingtan Song, Fengjun Liu, Dong Sun, Kexin Wang, Hui Qu

**Affiliations:** Department of General Surgery, Qilu Hospital, Shandong University, No.107, West Wenhua Road, Jinan, Shandong Province 250012 China; Department of General Surgery, Liaocheng Third People’s Hospital, Liaocheng, Shandong 252000 China; Department of General Surgery, Affiliated Hospital of Binzhou Medical University, Binzhou, Shandong 256603 China

**Keywords:** Gastric cancer, miR-152, TGF-β, HLA-G

## Abstract

**Background:**

Mounting evidences have showed the important role of transforming growth factor-β (TGF-β) in immunological surveillance of tumors. Some studies have also indicated human leukocyte antigen (HLA)-G-associated immune escape involving TGF-β management in gastric cancer (GC). However, the mechanism underlying it is unclear. This study aims to verify the correlations between HLA-G and TGF-β, involving the potential targeting of miR-152 on HLA-G.

**Results:**

TGF-β and HLA-G levels were analyzed in blood samples from twenty GC patients with ELISA assays, while TGF-β showed directly proportional to HLA-G levels in GC patients, and TGF-β induced HLA-G up-regulation was also confirmed in GC cell lines. Furthermore, miR-152 expression could be inhibited by TGF-β, and the negative post-transcriptionally regulation of miR-152 on HLA-G was also demonstrated through gain- and loss-of-function studies. Besides, miR-152 overexpression repressed HLA-G up-regulation induced by TGF-β. And, miR-152 expression levels showed inversely proportional to both HLA-G and also TGF-β levels in GC patients.

**Conclusion:**

TGF-β could induce HLA-G expression in GC by inhibiting miR-152, involving its negative regulation on HLA-G. Since TGF-β induced HLA-G up-regulation plays important role in immune escape, a potential application of miR-152 was suggested in GC treatment, or miR-152 might be one potential biomarker for GC.

## Background

Gastric cancer (GC) is the most frequent cause of cancer-related death, with an incidence of approximately 934,000 cases per year in East Asia [1]. Despite significant advances in diagnostic and therapeutic approaches during the last decades, the prognosis of GC also remains dismal because of its high recurrence and metastasis [2]. The pathogenesis of GC is multifactorial, and genetic alterations involving tumor suppressor genes, oncogenes, growth factors, and cell adhesion molecules are associated with the predisposition to GC [3]. Therefore, gaining a better understanding of the molecular mechanisms involved in GC is of great clinical significance.

MicroRNAs (miRNAs) are endogenously expressed noncoding RNAs that post-transcriptionally regulate expression of their target genes [4]. Increasing numbers of studies have shown aberrant expression of miRNAs presented in different types of cancers and have shown that miRNAs were involved in the regulation of the proliferation, differentiation, and apoptosis [5]. In GC, many miRNAs have been found to suppress metastasis, as well as help in the prediction of metastasis through their expression levels [6]. MiR-152 has been identified as a down-regulated miRNA in GC [7], and a recent study has revealed that miR-152 could suppress gastric cancer cell proliferation and motility by targeting CD151, which is an essential marker for the prognosis of GC due to the positive association with the invasiveness of GC [8]. However, the detailed role of miR-152 is still unclear.

Transforming growth factor-β (TGF-β) produced by T cells has been demonstrated as an important factor for suppressing antitumor immune responses, while increased TGF-β level has been reported in gastric cancer [9, 10]. Furthermore, TGF-β is reported to be critical for tumor progression and evasion from immune surveillance [11]. Although the mechanism of TGF-β in immune evasion is unclear, some studies have indicated that Human leukocyte antigen (HLA)-G is involved in it. Increased HLA-G level has been also reported in GC patients [12], and HLA-G-positive gastric cancers are often associated with poor survival [13]. Moreover, HLA-G associated immune escape in gastric cancer might be mediated by suppressing CD8+ T lymphocytes and increasing local Foxp3+ regulatory T (Treg) cells [14].

*In silico* analysis of microRNAs targeting the HLA-G 3'-UTR alleles and haplotypes has been performed with results indicating miR-152 targeting on HLA-G [15]. Besides, the relationship between miR-152 and HLA-G has also been investigated in human trophoblast cell line, which revealed that miR-152 could repress HLA-G expression and overexpression of miR-152 led to increased NK cell–mediated cytolysis, indicate that miR-152 may function as an immune system enhancer [16]. However, the modulation of miR-152 on HLA-G expression has not been proven yet in GC, let along relative mechanisms underlying TGF-β-induced immune escape. Our study found that HLA-G level is positively associated with TGF-β in GC patients. Therefore, we want to investigate whether HLA-G level was regulated by miR-152 expression in GC cells. Additionally, the regulated role of miR-152 was further confirmed in TGF-β-induced immune escape, as well as miR-152 expression in GC patients.

## Methods

### Patients and specimens

Paraffin-embedded, formalin-fixed tumor sections were obtained from twenty patients (15 males, 5 females) with gastric cancer that underwent surgical resection from September 2012 to July 2013 at the Qilu Hospital of Shandong University. Peripheral blood samples were collected from these patients one day before surgery. And, EDTA-plasma were prepared as described before [17] and frozen at -80 °C immediately for future use. None of the patients had received immunosuppressive drugs or chemotherapy before surgical resection. The study was approved by the Research Ethics Committee of Qilu Hospital of Shandong University, and written informed consents were obtained from these patients before participating in the study.

### Cell culture and treatment

Two human GC cell lines, BGC823 and SGC7901, were obtained from the Chinese Academy of Sciences (Shanghai, China). Cells were cultured in RMPI-1640 medium supplemented with 10 % FBS (Invitrogen, NY, USA) and antibiotics (1 % streptomycin/penicillin, Sigma-Aldrich) at 37 °C with 5 % CO_2_. After approximately 80 % confluence, the cells were starved for 24 h and treated without or with recombinant human TGF-β (R&D Systems, Minneapolis, MN) at various doses and for various durations according to the requirements.

### ELISA assays

EDTA-plasma samples or cell culture supernatants after 48 h incubation in serum-free medium were prepared for ELISA (Enzyme-Linked Immuno Sorbent Assays) analysis. TGF-β levels and soluble HLA-G concentrations were measured by use of a TGF-β ELISA kit (R&D Systems, Minneapolis, MN) and sHLA-G kit (Biovendor&Exbio, Praha, CZ) respectively according to the manufacturer’s instructions. Three independent experiments were repeated for each point.

### Transfection and luciferase assay

Double-stranded miR-152 mimics, single-stranded miR-152 inhibitor, or their relative negative control RNA (GenePharma, Shanghai, China) at a final concentration of 50 nM was introduced into cells. And, cells were transfected at approximately 70 % confluence using Lipofectamine 2000 (Invitrogen) in Opti-MEM medium (Invitrogen). PGL3-HLA-G was generated using the following primers for the amplification of HLA-G 3’UTR HLA-G-(3’UTR)-forward: 5’-ACTCTCATCGAGGGAGGAAA-3’; HLA-G-(3’UTR)-reverse: 5’-AAAGTTCTCATGTCTTCCATTT-3’. The PCR fragment was cloned into a firefly luciferase vector (pGL3; Promega, Madison, WI) within KpnI and XhoI restriction sites (Invitrogen). Mutation in the miR-152 binding-sites module of HLA-G 3’UTR was introduced by site-directed mutagenesis for the construction of Mut-HLA-G luciferase plasmid (PGL3-mut-HLA-G). Then, pGL3-HLA-G and PRL-TK Renilla luciferase plasmid were co-transfected, together with miR-152 mimics, inhibitor or negative miRNA (GenePharma) for luciferase reporter assay. After 24 h incubation, luciferase activity was measured using the Dual Luciferase Reporter 1000 Assay System (Promega), with results normalized to those of Renilla luciferase activities. All experiments were carried out at least in triplicate.

### RNA isolation and quantitative real-time PCR

Total RNA was isolated from cells and tissues using Trizol (Invitrogen). For the detection of the HLA-G mRNA, RNA was reverse transcribed into cDNA primed by oligo-dT primers using SuperScript III Reverse Transcriptase (Invitrogen), following the manufacturer’s instructions. GAPDH was used as an endogenous control. For the detection of mature miR-152, the enrichment of small RNA was carried out with the mirVana miRNA Isolation Kit (Ambion, Austin, TX). Small nuclear RNA U6 was used as an endogenous control. Quantitative real-time PCR (qRT-PCR) was performed on ABI 7300 SYBR Premix Ex Taq from Takara (Tokyo, Japan). Specific primers for HLA-G and GAPDH were as follows: HLA-G-forward: 5’-CTGGTTGTCCTTGCAGCTGTAG-3’, HLA-G-reverse: 5’-CCTTTTCAATCTGAGCTCTTCTTTCT-3’; GAPDH-forward: 5’-TCCACTGGCGTCTTCACCA-3’, GAPDH-reverse: 5’-CTGTGGTCATGAGTCCTTCC-3’. Primers for miR-152 and U6 were purchased from GeneCopoeia (Rockville, MD, USA). Data analysis was done by the ΔCT method for relative quantification.

### Statistical analysis

The results are presented as mean ± SD. Correlations were evaluated by Pearson’s correlation. Differences between groups were analyzed using a one-way ANOVA, Mann-Whitney *U* test or χ^2^ test, where appropriate. Statistical analyses were performed using SPSS 17.0 computer software (SPSS Inc., Chicago, IL). *P* < 0.05 was considered statistically significant.

## Results

### Clinical and demographic characteristics

Retrospective examination was performed of twenty GC patients who underwent surgery for gastric cancer from 2012 to 2013 at the Qilu Hospital of Shandong University. Patient characteristics are shown in Table 1.

**Table 1:** Characteristic of patients

Variable	Patients
Number of cases	20
Sex	
Male/Female	15/5
Age at diagnosis (mean)	58.9
Clinical pathology features	
TNM stage	
I	3
II	5
III	12
Lymph node metastasis	
Negative	5
Positive	15
Histologic grade	
Well	4
Poor	16

### TGF-β promoted the expression of HLA-G in GC

**Fig. 1:**
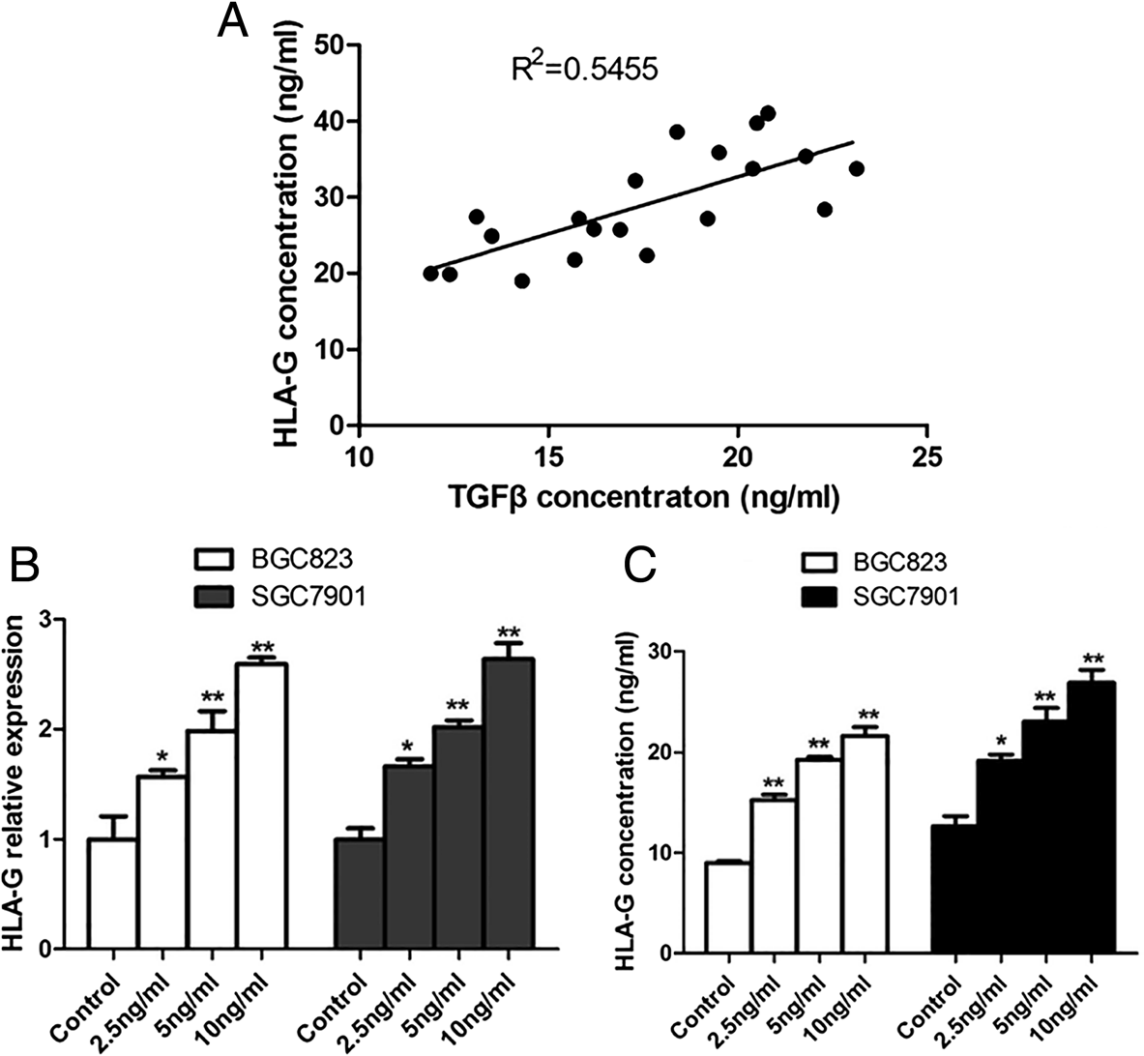
Correlation between TGF-β and HLA-G in GC. **a**. The concentration of TGF-β showed directly proportional to the HLA-G levels from the peripheral blood of twenty GC patients. R^2^ = 0.5455; *p* < 0.001. **b**. After treated with TGF-β (2.5, 5 or 10 ng/ml) for 24 h, HLA-G mRNA level was significantly upregulated in a dosage dependent manner in GC cell lines like BGC823 and SGC7901. **c**. ELISA analysis showed increased HLA-G level in the same manner after TGF-β treatment for 48 h. *P < 0.05; **P < 0.01

To elucidate the relationship between the levels of TGF-β and HLA-G in GC, their concentrations were analyzed in the peripheral blood samples from GC patients. A strong positive correlation between TGF-β and HLA-G levels evaluated by Pearson’s regression (R^2^ = 0.5455; *p* < 0.001) was noted in Fig. 1a. In order to investigate the relationship between TGF-β and HLA-G, two human GC cell lines, BGC823 and SGC7901, were co-incubated with TGF-β at various doses (2.5, 5 or 10 ng/ml). Then, the mRNA expression and supernatant level of HLA-G were detected after treated for 24 h and 48 h respectively. As shown in Fig. 1b and c, HLA-G level was significantly up-regulated under TGF-β treatment in a dosage dependent manner. Over 2.5-fold increase of HLA-G mRNA level was observed in both BGC823 and SGC7901 cells when treated with 10 ng/ml TGF-β (Fig. 1b), while soluble HLA-G concentration increased from approximately 10 ng/ml to 21 ng/ml or 12 ng/ml to 26 ng/ml in BGC823 and SGC7901 cell cultures respectively (Fig. 1c).

### MiR-152 expression was negatively regulated by TGF-β addition in GC cell lines

**Fig. 2:**
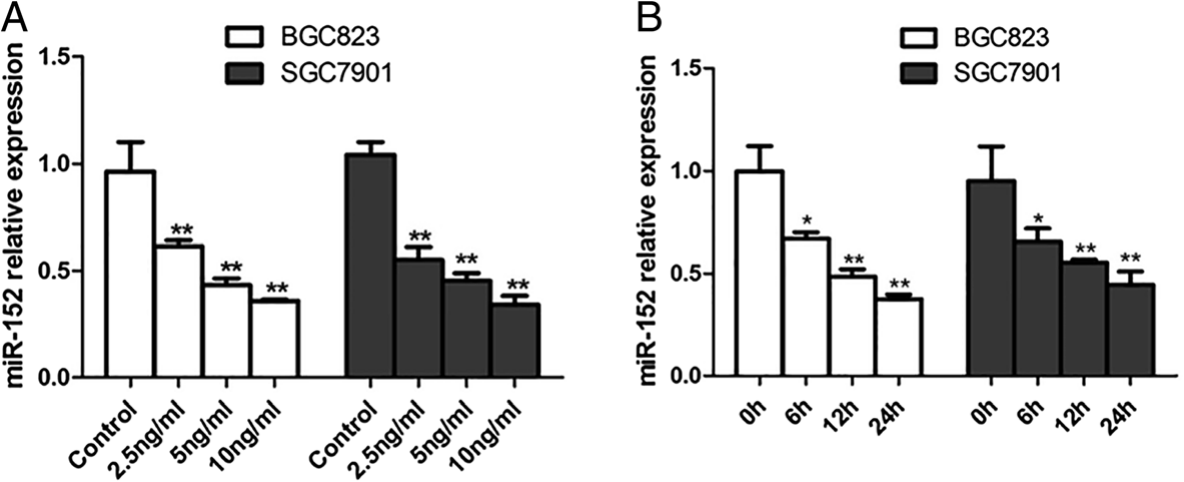
TGF-β inhibited miR-152 levels in GC cell line. The expression of miR-152 was downregulated under TGF-β treatment in dosage (2.5, 5 or 10 ng/ml for 12 h; **a**) and time (5 ng/ml for 6, 10 or 24 h; **b**) dependent manner. *P < 0.05; **P < 0.01

As a first step to reveal the functional role of miR-152, involving targeting on HLA-G, miR-152 expression was also investigated under TGF-β treatment at various doses (2.5, 5 or 10 ng/ml) and also for various durations (6, 12 or 24 h). As expected, miR-152 expression was significantly suppressed by TGF-β in both dosage and time dependent manner. Around 0.3-fold change of miR-152 expression was detected when treated with 10 ng/ml TGF-β for 12 h (Fig. 2a) or 5 ng/ml TGF-β for 24 h (Fig. 2b). Since increased HLA-G level was also observed under TGF-β treatment, it can be speculated that the potential targeting of miR-152 on HLA-G involved in TGF-β-induced up-regulation of HLA-G.

### MiR-152 showed negative targeting on HLA-G expression in GC cell lines

**Fig. 3:**
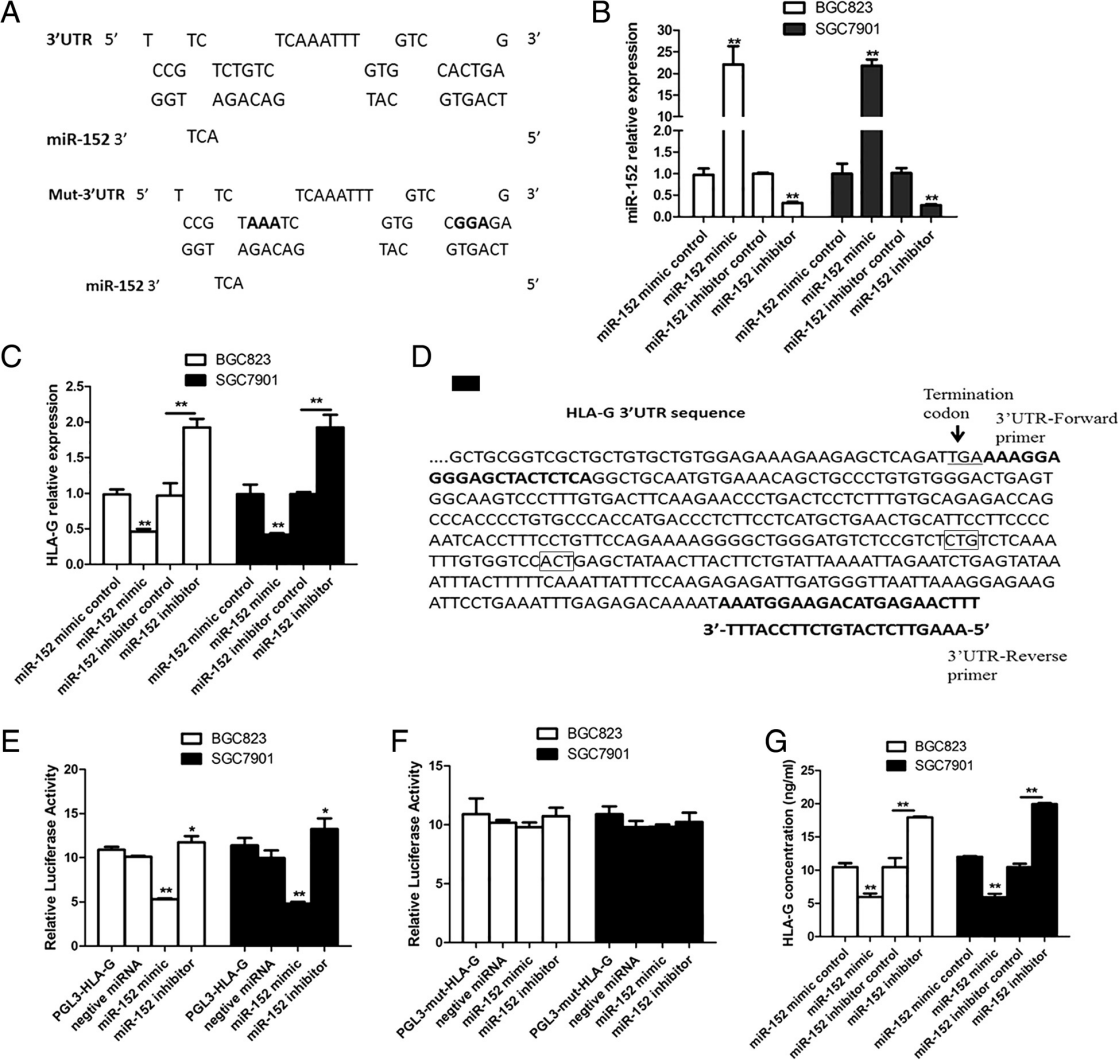
MiR-152 regulated HLA-G expressions in BGC823 and SGC7901 cells. **a**. The binding sites of miR-152 on the wild-type or mutated 3’UTR sequences of HLA-G, wherein “CTG” and “ACT” were mutated to “AAA” and “GGA” respectively in Mut-HLA-G. **b**. The expression change of miR-152 in BGC823 and SGC7901 cells after transfected with miR-152 mimic or inhibitor for 12 h. **c**. HLA-G mRNA expression in miR-152 overexpressing or inhibiting cells after transfection for 24 h. **d**. Primers used for the construction of HLA-G luciferase reporter (PGL3-HLA-G) within HLA-G 3'-UTR sequences. Mutation sites within Mut-HLA-G sequences were indicated with rectangle boxes. **e**. HLA-G 3’-UTR activity change resulted from miR-152 dysregulation through luciferase assay. The results were normalized to those of *Renilla* luciferase activities, and statistically analyzed as compared to that from the cotransfection with negative miRNA. **f**. Detection for Mut-HLA-G 3’-UTR activity change. **g**. HLA-G level detection after miR-152 mimics or inhibitor transfection for 48 h. *P < 0.05; **P < 0.01

In order to verify aforementioned speculation, the potential targeting of miR-152 on HLA-G was thoroughly investigated. Firstly, the binding sites of miR-152 on HLA-G 3’UTR was analyzed through sequence analysis, with derived Mut-HLA-G 3’UTR (Fig. 3a). The expression of miR-152 was confirmed after miR-152 mimics or inhibitor transfection for 12 h in both BGC823 and SGC7901 cells (Fig. 3b). After transfection for 24 h, HLA-G mRNA level showed significantly decrease or increase in miR-152 overexpressing or inhibiting cells, respectively (Fig. 3c). And, results from luciferase assay showed that HLA-G 3’UTR was also negatively regulated by miR-152, while no obvious regulation on Mut-HLA-G 3’UTR was observed at the same time (Fig. 3e and f). Moreover, similar trends of soluble HLA-G concentration were observed after transfection for 48 h (Fig. 3g), indicating that miR-152 suppressed HLA-G expression post-transcriptionally in GC cells.

### Inhibition of TGF-β on miR-152 expression contributed to induced HLA-G levels

**Fig. 4:**
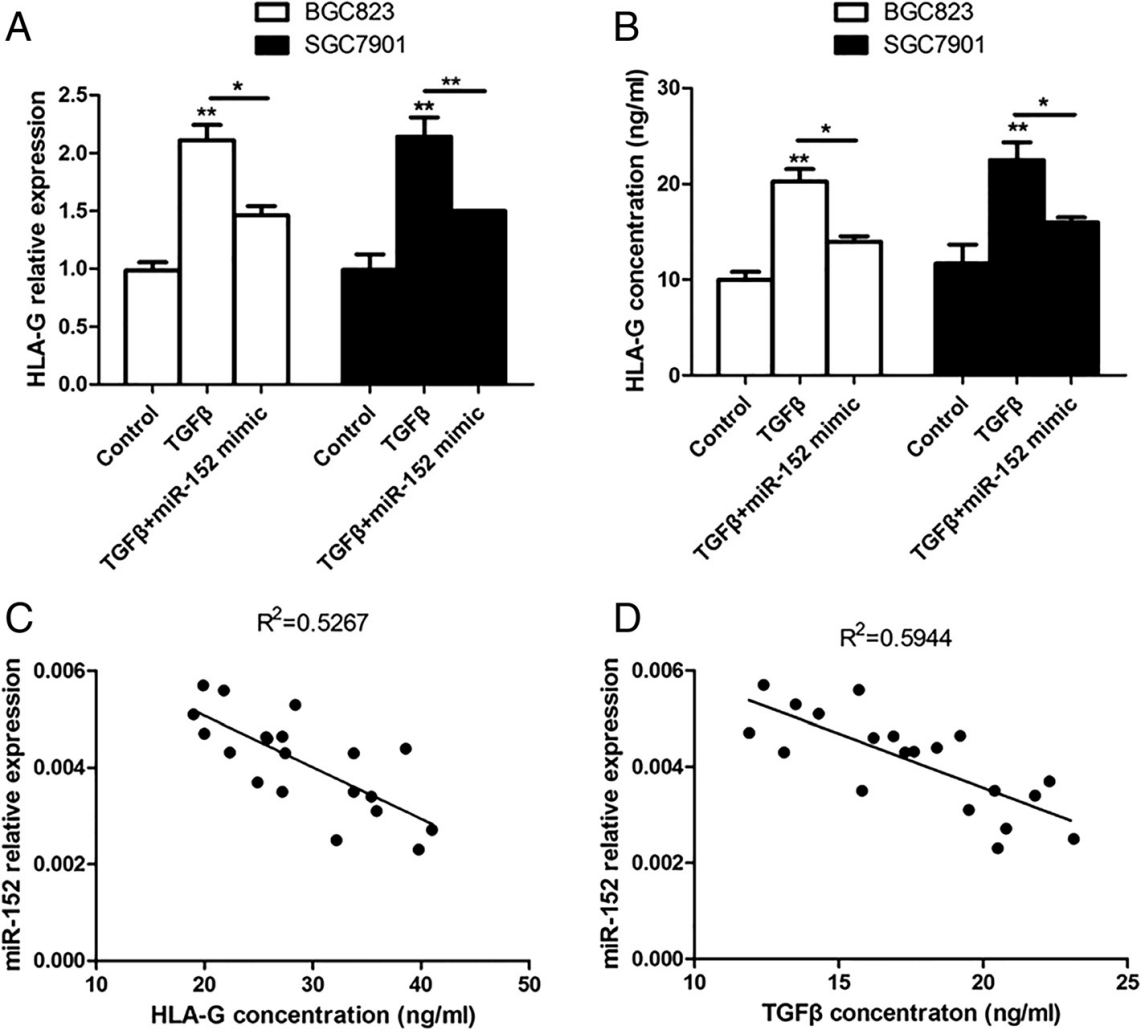
TGF-beta induced HLA-G expression by inhibiting miR-152. BGC823 and SGC7901 cells were transfected with miR-152 mimics for 12 h, then incubated for 12 h (for qPCR analysis) or 24 h (for ELISA analysis) and finally treated with TGF-β (5 ng/ml). TGF-β induced increase of mRNA level (**a**) or concentration (**b**) of HLA-G was attenuated by miR-152 overexpression. *P < 0.05; **P < 0.01. **c**. The expression of miR-152 in GC tissues was inversely proportional to the HLA-G levels of GC patients. R^2^ = 0.5267; *p* < 0.001. D. MiR-152 expression levels also showed inversely proportional to the concentration of TGF-β. R^2^ = 0.5944; *p* < 0.001

Further experiments were performed to investigate whether overexpression of miR-152 could attenuate the up-regulation of HLA-G induced by TGF-β. After transfected with miR-152 mimics for 12 h, BGC823 and SGC7901 cells were prepared for the treatment without or with TGF-β (5 ng/ml). As expected, TGF-β induced increase of HLA-G mRNA level was attenuated by miR-152 overexpression (Fig. 4a), as well as soluble HLA-G concentration (Fig. 4b). On the other hand, miR-152 expression was also detected in GC patients’ tissues. And, miR-152 expression levels showed inversely proportional to both HLA-G concentration (Fig. 4c) and also TGF-β levels (Fig. 4d). Taken together, it was concluded that TGF-β induced HLA-G expression by inhibiting miR-152, involving its negative regulation on HLA-G.

## Discussion

The TGF-β signalling pathway often plays a critical and dual role in the progression of human cancer [18]. During the early phase of tumor progression, TGF-β acts as a tumor suppressor, exemplified by deletions or mutations in the core components of the TGF-β signalling pathway. On the contrary, TGF-β also promotes processes that support tumor progression such as tumor cell invasion, dissemination, and immune evasion. Researchers have demonstrated that TGF-β is required for the induction and maintenance of regulatory T cells (Tregs) [19, 20], while Treg-mediated immunosuppression represents one of the crucial tumor immune evasion mechanisms in tumor progression [21]. Moreover, TGF-β and Tregs showed a positive correlation in circulation of GC patients, suggesting that TGF-β may contribute to the increase of Tregs in GC [22]. Therefore, TGF-β might act as an important factor for suppressing antitumor immune responses in GC progression.

HLA-G, a non-classic MHC class I protein, was first observed on extravillous cytotrophoblasts that play an important role in immune tolerance during pregnancy [23]. Studies have shown that HLA-G is detected in tumor tissue but rarely in normal tissue, suggesting its specific association with tumor growth and progression, for example by suppressing immune regulation, similar to maternal immune tolerance [24]. Many studies have reported the association between HLA-G-positive gastric cancer patient and poor survival [13], with a recent study suggesting the tumor escape mechanism by increasing Foxp3 + Treg lymphocytes and decreasing CD8 + T lymphocytes in GC [14]. HLA-G expression is also thought to contribute to the escape in immune surveillance by suppressing NK cell function [25], while TGF-β can also repress NK cell activity [18]. In the present study, we found a strong positive correlation between TGF-β and HLA-G in circulation of GC patients, while TGF-β induced HLA-G up-regulation was also demonstrated in GC cell lines.

Results from an *in silico* analysis of microRNAs targeting the HLA-G 3'-UTR suggest that some miRNAs might play a relevant role on the HLA-G expression pattern, including miR-152 [15]. MiR-152 belongs to miR-148/152 family that consists of miR-148a, miR-148b, and miR-152. Many studies have indicated that miR-152 potentially functions as a tumor suppressor and is down-regulated in various tumor types [26–28]. In GC, it was noted that both miR-148a and miR-152 were down-regulated in cancer tissue and cell lines, involving the cholecystokinin B receptor (CCKBR) protein [7]. In this study, we validated that HLA-G expression was repressed by miR-152 while miR-152 expression was also inhibited by TGF-β induction in GC cell lines. These results suggest that miR-152 might also play an essential role in GC immune escape. Further experiments showed that miR-152 overexpression could reversely repress TGF-β induced HLA-G up-regulation. Moreover, miR-152 expression level in GC tissues also showed strong negatively proportional to both TGF-β and HLA-G levels in circulation. Our work sheds new light on the regulation of GC progression by miR-152, involving HLA-G-associated immune escape regulated by TGF-β.

## Conclusions

In summary, this is the first study to show the positive correlation between TGF-β and HLA-G in circulation of GC patients, and we also found that miR-152 expression played a regulatory role in TGF-β induced HLA-G up-regulation. Further study could be executed to investigate the potential application of miR-152 in GC treatment.
